# Periadrenal mature cystic teratoma mimicking adrenal myelolipoma: a case report and review of the literature

**DOI:** 10.1093/jscr/rjag803

**Published:** 2026-09-25

**Authors:** Ran Hong

**Affiliations:** Department of Pathology, Medical School, Chosun University, 365 Philmundaero, Dong-gu, Gwangju, 61453, Korea (the republic of)

**Keywords:** adrenal region, periadrenal tumor, mature cystic teratoma, myelolipoma, retroperitoneal tumor

## Abstract

Mature teratomas arising in the adrenal region are rare and may mimic adrenal myelolipomas, particularly when adipose tissue is predominant. A 72-year-old woman with uncontrolled hypertension was found to have an 8.6-cm fat-containing mass in the right adrenal region, radiologically diagnosed as an adrenal myelolipoma. Laparoscopic adrenalectomy was performed. Histologically, the tumor predominantly consisted of mature adipose tissue with scattered epithelial-lined cystic structures, neural tissue, cartilage, thyroid tissue, bone marrow elements, and smooth muscle, confirming a mature cystic teratoma. Only a small amount of compressed adrenal tissue was identified at the tumor periphery without direct histologic continuity, supporting a periadrenal retroperitoneal origin. This case highlights that fat-dominant mature teratomas can closely mimic adrenal myelolipomas and should be considered in the differential diagnosis of heterogeneous fat-containing masses in the adrenal region.

## Introduction

Teratomas are germ cell tumors composed of tissues derived from two or more embryonic germ layers [1]. Although they most commonly arise in the gonads, extragonadal teratomas are rare and typically occur along the midline, including the mediastinum, sacrococcygeal region, and retroperitoneum [1]. Retroperitoneal teratomas are uncommon in adults [1, 2]. Tumors in the adrenal region are particularly rare, and determining whether they arise from the adrenal gland or the adjacent retroperitoneum may be difficult [2, 3]. Radiologically, these tumors often contain fat, cystic components, and calcification, thereby overlapping with other adrenal lesions, particularly myelolipoma [4, 5]. We report a fat-dominant mature cystic teratoma in the periadrenal region of an elderly woman that was initially interpreted as an adrenal myelolipoma, highlighting an important radiologic–pathologic diagnostic pitfall.

## Case presentation

**Figure 1:**
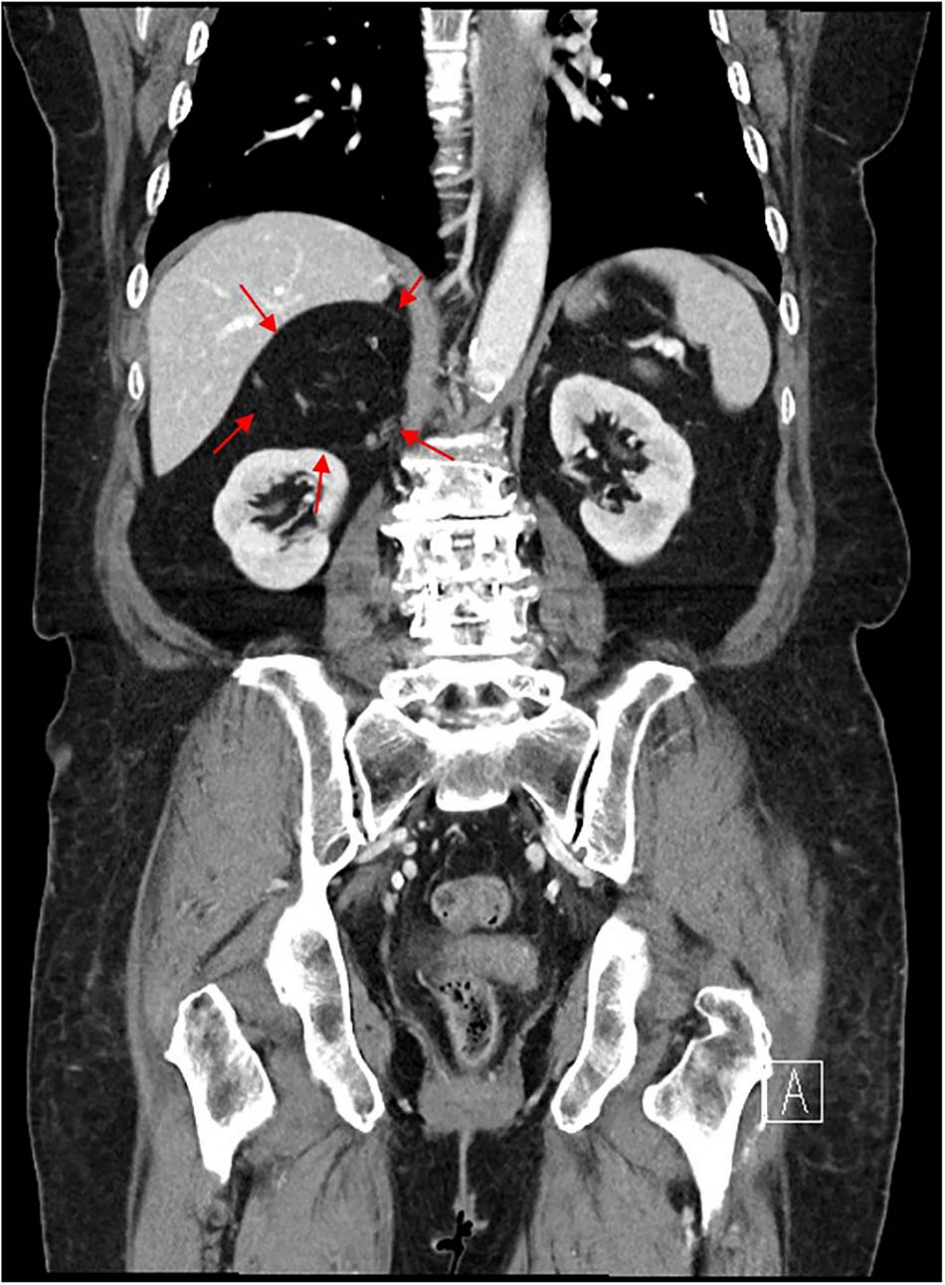
Contrast-enhanced abdominal CT showing a well-defined, fat-containing mass in the right adrenal region (arrows); the lesion demonstrates heterogeneous attenuation with focal soft-tissue components and was initially interpreted as an adrenal myelolipoma.

A 72-year-old woman presented with a 1-month history of severe hypertension, with blood pressure of ~200/120 mmHg. She had no significant medical history or symptoms suggestive of functional adrenal disease. Abdominal computed tomography (CT) revealed an ~8.6 cm, well-defined mass in the right adrenal region. The lesion predominantly showed fat attenuation with focal heterogeneous soft-tissue components (Fig. 1). Based on these findings, an adrenal myelolipoma was favored radiologically. Hormonal evaluation revealed no evidence of adrenal functional abnormality.

**Figure 2:**
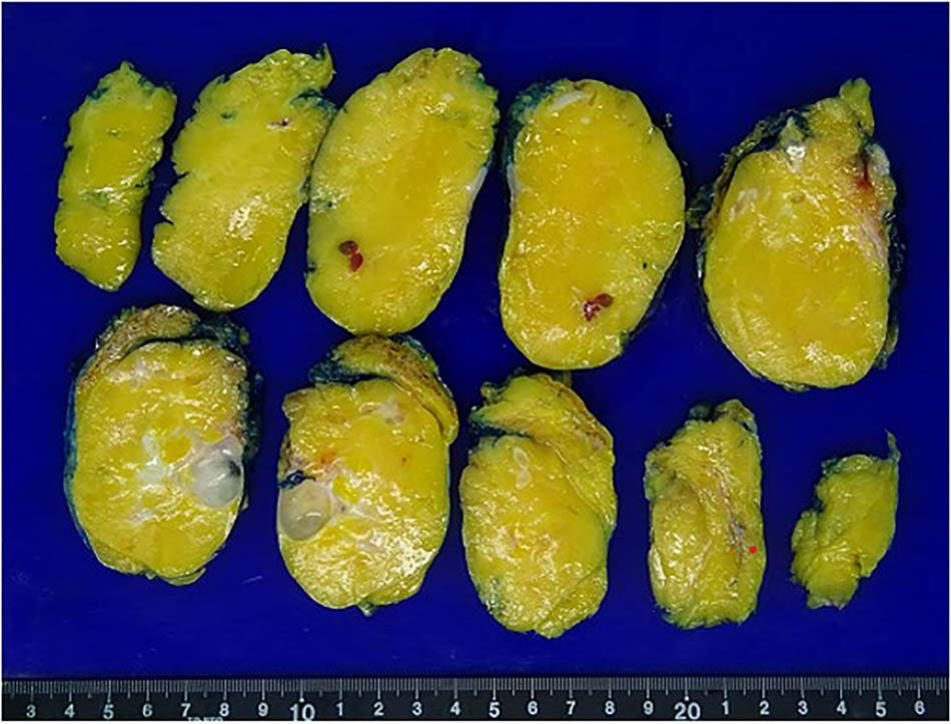
Gross appearance of the resected tumor; the tumor is composed predominantly of yellow, adipose-like tissue with focal cystic and hemorrhagic areas; the overall appearance closely resembles a lipomatous tumor.

**Figure 3:**
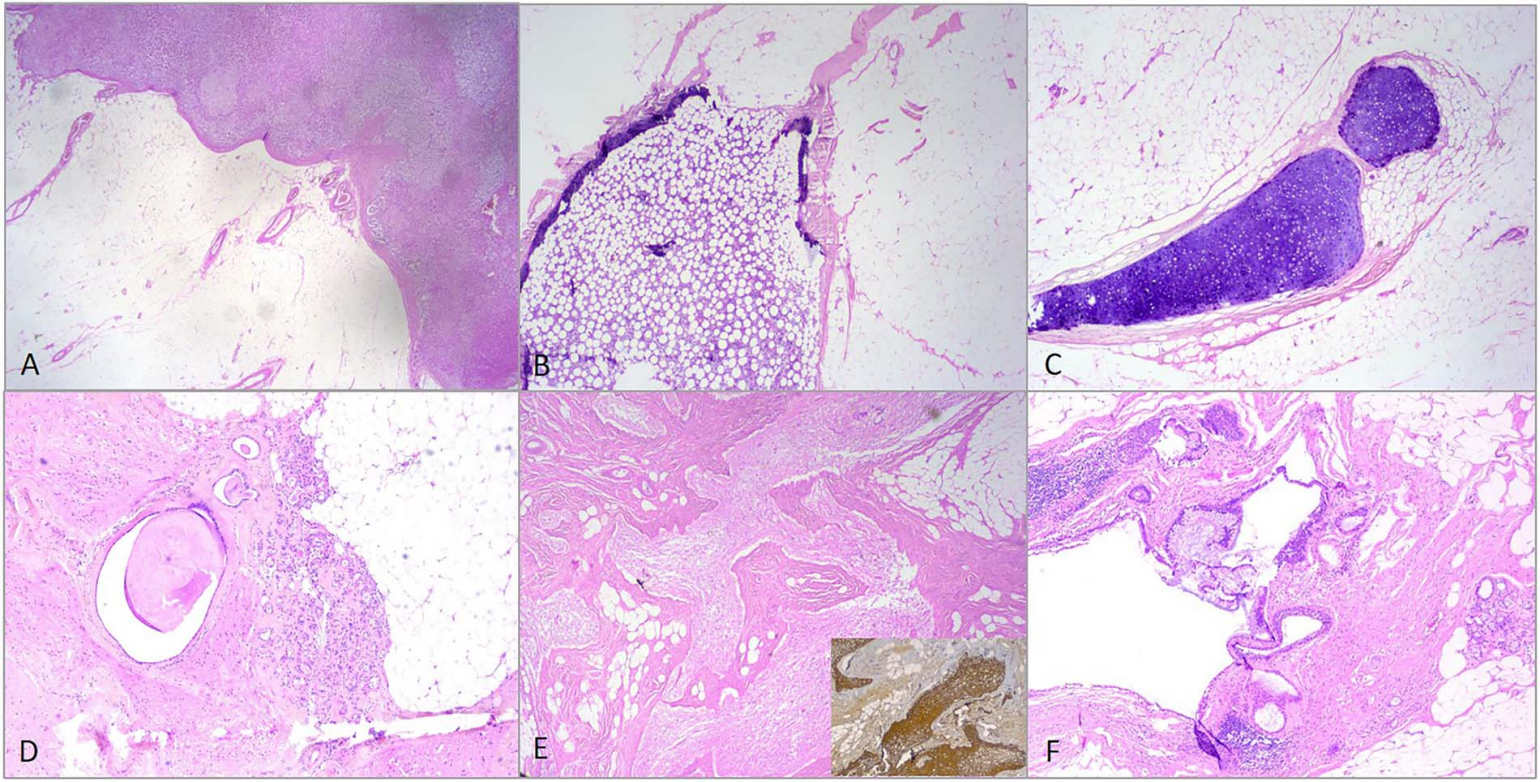
Histopathologic findings of the tumor; (A) a small focus of compressed adrenal tissue at the periphery of abundant adipose tissue (H&E, ×40); (B) bone marrow elements within adipose tissue (H&E, ×100); (C) mature cartilage (H&E, ×100); (D) thyroid tissue (H&E, ×100); (E) mature neural tissue resembling brain parenchyma (H&E, ×100); (F) an epithelial-lined cystic structure (H&E, ×100); the tumor is predominantly composed of adipose tissue with scattered mature elements derived from multiple germ layers, consistent with a mature cystic teratoma.

The patient underwent laparoscopic right adrenalectomy. Intraoperatively, the mass was located in close proximity to the adrenal gland and surrounding structures. Gross examination revealed a 9.0 × 8.5 cm well-circumscribed mass with predominantly yellow, adipose-like cut surfaces and focal cystic and hemorrhagic areas (Fig. 2). Microscopically, the tumor was composed predominantly of mature adipose tissue and contained scattered differentiated tissues, including epithelial-lined cystic structures lined by columnar, pseudostratified, and transitional-type epithelium, along with neural tissue, cartilage, thyroid tissue, bone marrow elements, and smooth muscle bundles (Fig. 3). No immature or malignant components were identified. Only a small amount of compressed adrenal tissue was present at the tumor periphery, without direct histologic continuity between the tumor and the adrenal cortex or medulla. These findings supported a diagnosis of mature cystic teratoma arising in the periadrenal retroperitoneal region.

The postoperative course was uneventful, and no recurrence was observed at 5 months. This study was approved by the Institutional Review Board of Chosun University Hospital (Gwangju, Republic of Korea), which waived the requirement for written informed consent because of the retrospective nature of the study (IRB No. 20226-37).

## Discussion

Teratomas are neoplasms derived from pluripotent germ cells and may contain a wide variety of mature tissues, including skin, neural tissue, cartilage, bone, and glandular epithelium [3, 6]. Although most mature teratomas are benign, malignant transformation has rarely been reported [1, 3]. Adrenal or periadrenal teratomas are extremely rare, with fewer than 70 cases described in the literature [1]. They show a slight female predominance and are more frequently detected in young adults, although occasional cases have been reported in elderly patients [1]. Most are nonfunctional and are discovered incidentally [1, 3].

A major diagnostic challenge is distinguishing a primary adrenal teratoma from a retroperitoneal teratoma arising adjacent to the adrenal gland [2, 3]. In many reported cases, imaging suggests an adrenal origin; however, histopathologic examination may demonstrate only compressed or displaced adrenal tissue without direct continuity with the tumor, favoring a periadrenal origin [2]. In the present case, only a small amount of compressed adrenal tissue was identified at the periphery of the mass, and no direct histologic continuity with the adrenal cortex or medulla was present. We therefore considered the lesion to be a periadrenal retroperitoneal teratoma rather than a primary adrenal teratoma.

Teratomas in the adrenal region typically appear as heterogeneous masses containing fat, cystic components, and occasionally calcification [3, 4]. Myelolipoma is the most common fat-containing adrenal mass; however, heterogeneous soft-tissue or cystic components should prompt consideration of alternative diagnoses [4, 5]. The present tumor was predominantly composed of mature adipose tissue and therefore closely resembled a lipomatous adrenal lesion both radiologically and grossly. In addition, the presence of bone marrow elements may have further contributed to the radiologic impression of myelolipoma. Careful histologic examination, however, revealed scattered mature tissues from multiple germ layers, including epithelial-lined cystic structures, neural tissue, cartilage, and thyroid tissue, establishing the diagnosis of mature teratoma. The correspondence between the heterogeneous imaging features and the diverse histologic components underscores the importance of radiologic–pathologic correlation.

Complete surgical excision is the treatment of choice and provides both definitive diagnosis and curative treatment [3, 4]. The prognosis of completely excised mature teratomas is generally excellent [3]. This case emphasizes that a fat-containing mass in the adrenal region should not automatically be regarded as a myelolipoma. Fat-dominant mature teratoma, although rare, should be included in the differential diagnosis when a lesion demonstrates heterogeneous non-adipose components.

## Conclusion

We report a rare fat-dominant mature cystic teratoma arising in the periadrenal region of an elderly woman and initially interpreted as an adrenal myelolipoma. Histologic demonstration of diverse mature tissues and the absence of direct continuity with the adrenal gland supported a periadrenal retroperitoneal origin. This case highlights a diagnostic pitfall in the evaluation of heterogeneous fat-containing masses in the adrenal region.
